# Behavioral and Disease-Related Characteristics of Patients with Acute Stroke during the Coronavirus Disease Pandemic

**DOI:** 10.3390/healthcare10040604

**Published:** 2022-03-23

**Authors:** Dougho Park, Eunhwan Jeong, Su Yun Lee, Mansu Kim, Dae Young Hong, Heum Dai Kwon, Mun-Chul Kim

**Affiliations:** 1 Department of Rehabilitation Medicine, Pohang Stroke and Spine Hospital, Pohang 37659, Korea; parkdougho@gmail.com; 2 Department of Neurology, Pohang Stroke and Spine Hospital, Pohang 37659, Korea; jeh132000@hanmail.net (E.J.); sunyu804@gmail.com (S.Y.L.); 3 Department of Neurosurgery, Pohang Stroke and Spine Hospital, Pohang 37659, Korea; pseudo1114@naver.com (M.K.); hongdy2000@gmail.com (D.Y.H.); spinekwon@gmail.com (H.D.K.)

**Keywords:** COVID-19, pandemic, stroke, healthcare surveys, Korean Stroke Registry

## Abstract

This study aimed to evaluate the behavioral and disease-related characteristics of patients with acute stroke during the Coronavirus disease (COVID-19) pandemic. This retrospective study was conducted using the Korean Stroke Registry database from a single cerebrovascular specialty hospital. We categorized the COVID-19 pandemic (February 2020 to June 2021) into three waves according to the number of COVID-19 cases recorded and the subjective fear index of the general population and matched them with the corresponding pre-COVID-19 (January 2019 to January 2020) periods. The total number of acute stroke hospitalizations during the pre-COVID-19 and COVID-19 periods was 402 and 379, respectively. The number of acute stroke hospitalizations recorded during the regional outbreak of COVID-19 was higher than that recorded during the corresponding pre-COVID-19 period (97 vs. 80). Length of hospital stay was significantly longer during the COVID-19 pandemic than during the pre-COVID-19 period (11.1 and 8.5 days, respectively; *p* = 0.003). There were no significant differences in the time from onset to hospital arrival, rate of acute intravenous/intra-arterial (IV/IA) treatments, and door-to-IV/IA times between the pre-COVID-19 and COVID-19 periods. This study suggests that specialty hospitals can effectively maintain the quality of healthcare through the management of acute time-dependent diseases, even during pandemics.

## 1. Introduction

Since its first report in late 2019, the coronavirus disease (COVID-19) pandemic has brought about changes in healthcare systems worldwide [1,2]. These changes have posed unexpected challenges, especially in the management of diseases that require timely treatment in the acute phase, such as stroke [3,4]. Studies conducted in several countries have indicated that stroke hospitalizations decreased during the COVID-19 pandemic [5,6,7]. South Korea has experienced multiple COVID-19 pandemic waves since the first local infection was confirmed on 30 January 2020 [8]. Since then, the distribution and utilization of medical resources in South Korea have been greatly affected. Jeong et al. [9] conducted a multicenter study to analyze the differences in the health-seeking behaviors of patients with acute stroke between 18 February 2020 and 31 March 2020, the period of the first COVID-19 outbreak in South Korea. The results showed a decrease in the number of patients with acute stroke and an increase in time from onset to hospital arrival during the first COVID-19 outbreak.

Regarding South Korea’s medical delivery system, there have been long-standing problems of regional imbalance in the distribution of medical institutions and the phenomenon of concentration in tertiary university hospitals or large-scale general hospitals [10]. Acute stroke is not exempt from this phenomenon [11]. The management of acute stroke requires well-trained staff and intensive support from related fields because of its high level of difficulty. Therefore, it is difficult for medium- or small-sized hospitals to establish an integrated stroke care system [12]. Further, due to the time-dependent characteristics of acute stroke, inequity in the regional distribution of stroke centers has been recognized and taken more seriously [12,13]. Considering these challenges, the Korean Ministry of Health and Welfare has been designating and maintaining specialty hospitals since 2011 to provide specialized high-level medical services, prevent concentration in tertiary medical institutions, and evenly distribute the available medical resources [14]. Hospitals are designated as specialty hospitals if they meet all the prerequisite standards regarding patient composition ratio, facilities, and human resources presented by the Health Insurance Review and Assessment service [15]. Cerebrovascular specialty hospitals can be a solution to the abovementioned problems regarding the management of acute stroke. Cerebrovascular specialty hospitals are required to perform qualified open cranial surgery and provide appropriate acute intravascular intervention and integrated stroke care in a competent and timely manner. Currently, there are four cerebrovascular hospitals in South Korea [16].

Several multicenter reports on COVID-19-related changes in the volume of medical utilization by patients with acute stroke have been published [7]. However, only a few of such reports are from cerebrovascular specialty centers. In addition, most of the previous studies focused on the early pandemic period (February 2020 to March 2020) [17]. Therefore, the aim of the present study was to evaluate and compare the behavioral patterns and disease-related characteristics of patients with acute stroke during the pre-COVID-19 and COVID-19 periods using data from a cerebrovascular specialty hospital in South Korea. We assumed that the specialized center would maintain its original function even in the case of a pandemic and aimed to test this hypothesis in patients with acute stroke admitted to a cerebrovascular specialty hospital.

## 2. Materials and Methods

### 2.1. Participants, Data Source, and Ethics Statements

This was a retrospective study conducted from January 2019 to June 2021. Patients with acute stroke admitted to a single cerebrovascular specialty hospital in South Korea were included in this study. All the patients included in this study were enrolled in the Korean Stroke Registry (KSR; www.strokedb.or.kr; accessed on 3 December 2021), a multicenter, prospective, hospital-based registry system. All patients provided informed consent before enrolment into the KSR (PSSH0475-201901-HR-001). The study design was reviewed and approved by the institutional review board of Pohang Stroke and Spine Hospital (PSSH0475-202112-HR-021-01). Informed consent for this study was waived owing to the retrospective nature of the study and the anonymity of the KSR database. This study was conducted in accordance with the principles of the Declaration of Helsinki.

### 2.2. Study Design

**Figure 1:**
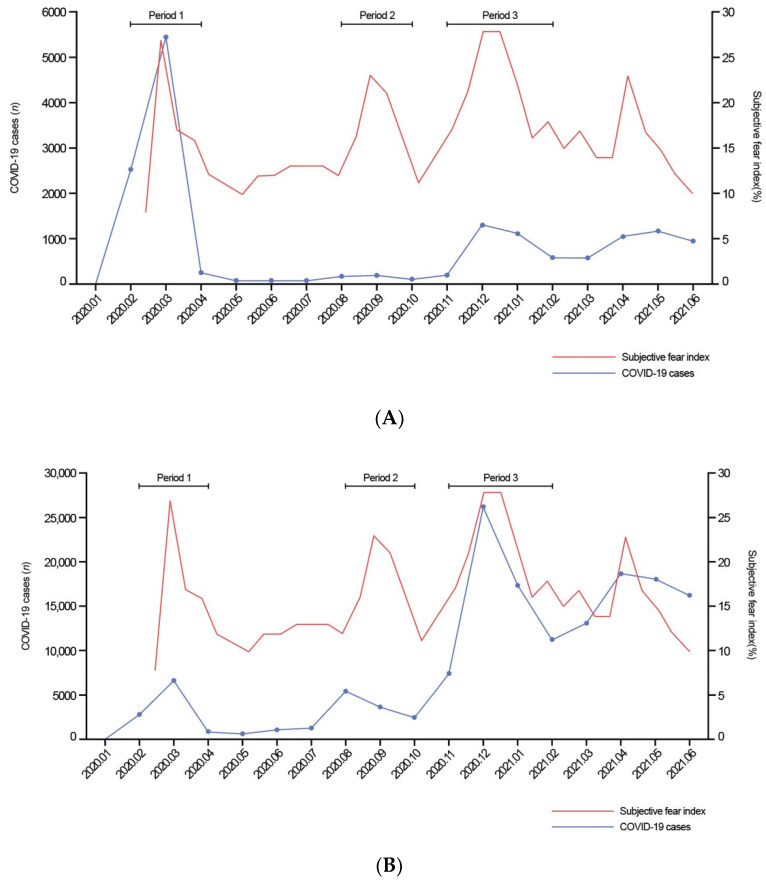
Definition of coronavirus disease (COVID-19) pandemic periods according to the (**A**) regional and (**B**) nationwide outbreaks.

The period from January 2019 to January 2020 was defined as the pre-COVID-19 period, whereas February 2020 to June 2021 was defined as the COVID-19 period. The COVID-19 period was stratified into three pandemic waves that were matched with the corresponding pre-COVID-19 periods in consideration of the seasonal variation in the incidence of stroke. The pandemic waves were defined based on the number of positive COVID-19 cases recorded and the fear index of infection in the general population. The number of positive COVID-19 cases recorded over the pandemic period was obtained from the Central Disease Control Headquarters of South Korea (ncov.mohw.go.kr/en; accessed on 1 December 2021). We referred to the results of a biweekly questionnaire survey provided by Hankook Research for the subjective fear index of COVID-19 infection in the general population (hrcopinion.co.kr/archives; accessed on 1 December 2021; only available in Korean). We checked the percentage of the survey participants who answered ‘high’ or ‘extremely high’ to the question ‘How likely do you think you are to be infected with COVID-19?’ Based on these two parameters, we defined the first pandemic wave (Period 1) as the period from February 2020 to March 2020, which was when a COVID-19 outbreak occurred in the region of Pohang City (Daegu metropolitan city and the surrounding Gyeongsangbuk-do province). The corresponding pre-COVID-19 period was defined as February 2019 to March 2019. We defined the second pandemic wave (Period 2) as a period from August 2020 to September 2020 and the corresponding pre-COVID-19 period from August 2019 to September 2019. Finally, we defined the third pandemic wave (Period 3) as a period from November 2020 to January 2021 and the corresponding pre-COVID-19 period from November 2019 to January 2020 (Figure 1).

**Figure 2:**
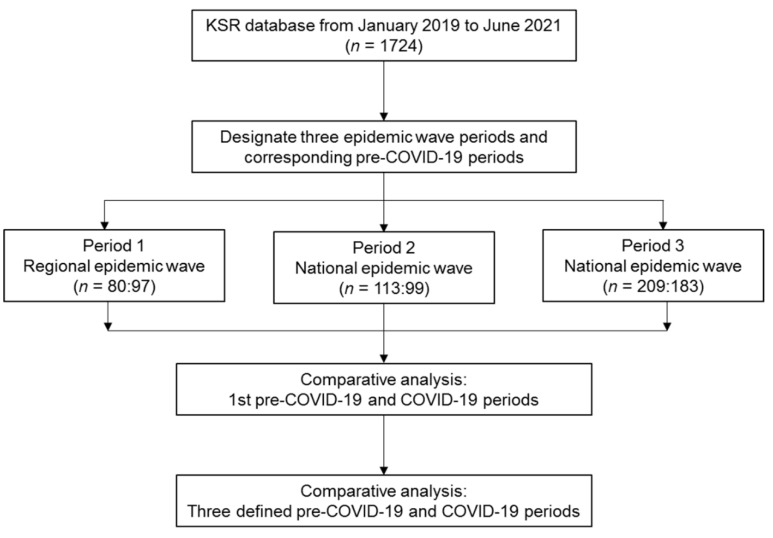
Flowchart of this study. KSR—Korean Stroke Registry; COVID-19—coronavirus disease.

Then, we conducted a comparative analysis of patients with acute stroke during the first pre-COVID-19 period and the first COVID-19 period (the regional outbreak period) and during all the defined pre-COVID-19 and COVID-19 periods (Figure 2).

The exclusion criteria for this study were: (1) patients who visited the hospital seven days or more after the onset of stroke; (2) patients with missing data; and (3) patients hospitalized for acute stroke but not enrolled in the KSR (overall acceptance rate: 94.9%).

### 2.3. Definitions of Variables

Age, sex, and body mass index were obtained as primary patient information. The premorbid functional level of each patient was evaluated using the modified Rankin scale. We also identified vascular risk factors, such as a previous cerebrovascular attack, coronary heart disease, hypertension, diabetes, dyslipidemia, atrial fibrillation, and current smoking status.

Regarding health-seeking-related factors, we identified the number of stroke patient admissions per month and its ratio to the total number of hospital admissions during the defined pre-COVID-19 and COVID-19 periods. In addition, the rates of admission of patients with clear onset stroke and the onset-to-arrival times (or last normal time) for the defined periods were confirmed. Patient admission routes were defined as an outpatient clinic, emergency department, and in-hospital. The length of hospital stay was also estimated.

As stroke-related factors, stroke subtypes (ischemic, hemorrhagic, and transient ischemic attack) were identified. We also confirmed the initial and discharge National Institutes of Health Stroke Scale (NIHSS) scores of the patients. For patients with ischemic stroke, subtypes were categorized according to the Trial of Org 10,172 in Acute Stroke Treatment classification. We also determined the rate of acute intravenous/intra-arterial (IV/IA) treatments and door-to-needle or puncture times.

### 2.4. Statistical Analysis

Continuous variables are expressed as median (interquartile range) and categorical variables as frequency (proportion). The normality of continuous variables was evaluated using the Shapiro–Wilk test. For the comparative analysis of patients with acute stroke during the pre-COVID-19 and COVID-19 periods, the Wilcoxon rank-sum test was used to assess continuous variables, whereas the chi-squared (trend) and Fisher’s exact tests were used for categorical variables. The Wilcoxon signed-rank test was used to compare the monthly acute stroke hospitalizations and their ratio for each period. Statistical significance was set at *p* < 0.05. All statistical analyses were performed using R software version 4.1.2 (R Core Team, R Foundation for Statistical Computing, Vienna, Austria).

## 3. Results

### 3.1. The Entire Pandemic Period (Periods 1, 2, and 3)

The total number of acute stroke hospitalizations recorded during the pre-COVID-19 and COVID-19 periods was 402 and 379, respectively. There was no significant difference in the number and rate of monthly acute stroke hospitalizations between the pre-COVID-19 and the COVID-19 periods. However, the length of hospital stay showed a significant difference between the two periods. The length of hospital stay was 11.1 days (range, 6.5–21.9) during the COVID-19 period, which is significantly longer than the 8.5 days (range, 5.4–17.5) during the pre-COVID-19 period (*p* = 0.003). Regarding patient comorbidities, the number of cases of coronary heart disease and dyslipidemia was significantly higher during the COVID-19 period, 11.6% and 66.8%, respectively, than during the pre-COVID-19 period, 7.0% and 56.5%, respectively (*p* = 0.034 and *p* = 0.004, respectively). There was no significant difference between the two periods in terms of discharge NIHSS score or mortality rate during hospitalization (Table 1).

Subgroup analysis of patients with ischemic stroke showed a significant difference between the subtypes of stroke recorded during the pre-COVID-19 and the COVID-19 periods. The rates of occurrence of large artery atherosclerosis and small-vessel occlusion were relatively low, whereas the rate of cardiac embolism was higher during the COVID-19 period than during the pre-COVID-19 period (*p* = 0.037). There were no significant differences between the two periods in terms of the rate of acute IV/IA treatments and door-to-IV/IA times (Table 2).

### 3.2. The Regional Outbreak (Period 1)

We also performed a comparative analysis of the first pre-COVID-19 period and the first COVID-19 period (Period 1, the regional outbreak period). A notable result was that the total number of acute stroke hospitalizations recorded during Period 1 (97) was higher than that recorded during the corresponding pre-COVID-19 period (80). Additionally, there was a significant difference in the admission route between the two periods. During Period 1, the rate of hospitalization in outpatient clinics was high, whereas the rate of hospitalization in the emergency departments was relatively low (*p* = 0.003). However, no significant difference in length of hospital stay was observed between Period 1 and the corresponding pre-COVID-19 period. In addition, there were no significant differences between Period 1 and the corresponding pre-COVID-19 period in terms of outcome variables (discharge NIHSS score and mortality rate during hospitalization) (Table 3).

Furthermore, there was no significant difference in stroke subtype and acute IV/IA treatment rates between Period 1 and the corresponding pre-COVID-19 period (Supplementary Table S1).

## 4. Discussion

It is well known that pandemics affect acute stroke care and are a burden on the stroke delivery system [6,18]. This study was conducted to evaluate the behavioral and disease-related characteristics of patients with acute stroke during the COVID-19 pandemic using a dataset from a cerebrovascular specialty hospital. Through this, we primarily intended to examine the role of specialized medical institutions during the pandemic. We found that the volume of acute stroke hospitalizations and acute IV/IA treatment during the COVID-19 pandemic did not decrease compared to the corresponding pre-COVID-19 period. Meanwhile, significant differences were noted in the length of hospital stay during the entire pandemic period and in the ratio of admissions through outpatient clinics during the initial COVID-19 outbreak period.

Regarding the length of hospital stay, there was no significant difference between Period 1 and the corresponding pre-COVID-19 period; however, significantly longer days were recorded during all the pandemic waves than during the corresponding pre-COVID-19 periods. This result was attributed to strict hospitalization standards and separation pathways applied by hospitals receiving transfers after acute stroke management (rehabilitation or convalescent hospitals) as the COVID-19 pandemic continued. During the initial pandemic period, there were relatively few restrictions on the transfer system between hospitals. However, after mass infections were recorded in several hospitals, negative polymerase chain reaction COVID-19 results were generally required before admission to rehabilitation or convalescent hospitals. In addition, it was challenging to find a referral hospital for patients with fever and other infectious symptoms unrelated to COVID-19 during the outbreak period. We surmised that these changes were important causes of delayed discharge of patients during the pandemic period. Meanwhile, the relatively increased proportion of hospitalizations via outpatient clinics during the first outbreak period might have been influenced by poor accessibility to the emergency department due to the complex triage system [19]. We also inferred that the vague anxiety of patients and their families regarding possible exposure to COVID-19 in the emergency department affected their accessibility to the facility [3,20].

The present study is remarkable because it is a stroke-related health-seeking behavior study based on experiences from a cerebrovascular specialty hospital during the COVID-19 pandemic. Of note, despite the regional outbreak or long pandemic wave, there was no decrease in the volume of stroke hospitalizations or in hospital utilization according to stroke type or severity during the pandemic period. Furthermore, there was no decrease in the proportion of acute IV/IA treatments and no delay in the door-to-vascular access time for patients with ischemic stroke during the COVID-19 pandemic waves. These results are inconsistent with those of previous related studies. Rudilosso et al. [21] analyzed and compared the characteristics of patients with acute stroke in March 2019 and March 2020 using data from the stroke center of a tertiary university hospital in Barcelona. They reported a significant decrease in the number of acute stroke admissions and thrombectomy cases and found that the ages of patients with stroke were significantly lower during the COVID-19 pandemic period. Based on Polish national data, Miękisiak et al. [22] reported a more than 8% reduction in stroke hospitalization and up to 15% reduction in thrombolysis during the COVID-19 pandemic. In another study conducted using the Chinese stroke registration dataset from 280 hospitals, Zhao et al. [23] reported an approximately 40% reduction in the number of stroke admissions and a 25% reduction in the number of thrombolysis/thrombectomy cases in February 2020. In addition, a multicenter study conducted in Germany showed that the number of stroke admissions decreased during the COVID-19 outbreak, whereas the rate of acute reperfusion therapy was not significantly affected [24]. In contrast, Sobolewski et al. [25] reported that the reperfusion procedure increased as the stroke with large vessel occlusion increased during the second wave of the COVID-19 pandemic in Poland. The present study did not show these reductions reported in previous studies. This suggests that our facility maintained its primary function as a regional hospital specialized in the management of cerebrovascular diseases, which was relatively unaffected by the COVID-19 pandemic. From the start of the pandemic, the original functions of tertiary or public hospitals were inevitably and considerably dispersed, as COVID-19-related studies have already demonstrated [26,27]. However, some specialized medical centers have the advantage of being relatively free from massive increases in services related to the pandemic [28]. We considered that our extensive experiences as a specialty hospital and the residents’ awareness of specialty hospitals made it possible for patients and their families to access the hospital without hesitation during an infectious disease outbreak that requires social distancing and isolation. Consequently, the results of our single-center study are significant because they demonstrate that fostering specialized hospitals can facilitate the efficient maintenance of the quality of acute time-dependent disease management, even during a pandemic [29].

This study had some limitations. First, as this was a single-center study, it is difficult to generalize its results to all regions and hospital types. However, our study was remarkable as it provided data from the perspective of a specialty hospital. Second, we presented objective results using the KSR database. However, not all patients with acute stroke are enrolled in the KSR. Although patients who are not enrolled in the KSR were not included in the present study, we do not believe this significantly impacted our results because the KSR rejection rate is just over 5%. Third, we did not present the long-term follow-up data of the patients. Finally, although South Korea is currently experiencing its fourth and largest wave of the COVID-19 pandemic, we cannot provide additional data obtained during this ongoing wave. Thus, further research is needed to identify the characteristics of patients with acute stroke during the fourth pandemic wave.

## 5. Conclusions

This study, which was conducted using data from a single cerebrovascular specialty hospital in South Korea, demonstrated that the volume of acute stroke hospitalizations and acute IV/IA treatment during the COVID-19 pandemic did not decrease compared to the corresponding pre-COVID-19 period. In addition, there were no delays in hospital arrival or administration of acute treatment during the COVID-19 period. The findings of this study suggest that the activation of specialized hospitals can effectively maintain the quality of the healthcare system for the management of acute time-dependent diseases, even during a pandemic. A multicenter and multinational future study is needed to verify these findings across various healthcare systems.

## Figures and Tables

**Table 1:** Demographic and disease-related characteristics of patients with stroke during the COVID-19 and pre-COVID-19 periods.

	Pre-COVID-19 (*n* = 402)	COVID-19 (*n* = 379)	*p* Value
Acute stroke cases, *n* (Monthly)	60.0 (42.0–73.0)	53.0 (49.0–56.0)	0.547
The ratio of acute stroke admission ^a^, %	5.3 (4.0–6.8)	5.6 (4.8–6.3)	0.578
Age, years	68.0 (58.0–77.0)	68.0 (59.0–79.0)	0.319
Male, *n* (%)	230 (57.2)	225 (59.4)	0.591
Previous mRS, *n* (%)			0.193
0	376 (93.5)	362 (95.5)	
1	17 (4.2)	9 (2.4)	
2	7 (1.7)	5 (1.3)	
3	2 (0.5)	0 (0.0)	
4	0 (0.0)	2 (0.5)	
5	0 (0.0)	1 (0.3)	
Clear onset type, *n* (%)	330 (82.1)	298 (78.6)	0.259
Time-to-arrival, hours			
Onset-to-arrival (*n* = 330:298)	12.4 (2.1–50.0)	12.4 (2.3–37.3)	0.797
LNT-to-arrival (*n* = 72:81)	14.3 (8.3–25.7)	14.6 (8.6–22.9)	0.808
Admission route, *n* (%)			0.991
Outpatient	51 (12.7)	49 (12.9)	
Emergency department	348 (86.6)	327 (86.3)	
In-hospital	3 (0.7)	3 (0.8)	
Stroke type, *n* (%)			0.856
Ischemic	297 (73.9)	275 (72.6)	
Hemorrhagic	83 (20.6)	80 (21.1)	
Transient ischemic attack	22 (5.5)	24 (6.3)	
Initial NIHSS	2.0 (1.0–5.0)	2.0 (1.0–6.0)	0.222
Vascular risk factor, *n* (%)			
Previous CVA	54 (13.4)	53 (14.0)	0.905
Coronary heart disease	28 (7.0)	44 (11.6)	0.034
Hypertension	237 (59.0)	237 (62.5)	0.342
Diabetes	107 (26.6)	100 (26.4)	>0.999
Dyslipidemia	227 (56.5)	253 (66.8)	0.004
Atrial fibrillation	46 (11.4)	56 (14.8)	0.202
Current smoker	102 (25.4)	110 (29.0)	0.286
Length of stay, days	8.5 (5.4–17.5)	11.1 (6.5–21.9)	0.003
Discharge NIHSS	1.0 (0.0–3.0)	1.0 (0.0–4.0)	0.104
Expired, *n* (%)	12 (3.0)	18 (4.7)	0.264

COVID-19—coronavirus disease; mRS—modified Rankin scale; LNT—last normal time; NIHSS—National Institute of Health Stroke Scale; CVA—cerebrovascular attack. ^a^ (the number of acute stroke admissions/the number of total admissions) ×100.

**Table 2:** Features of ischemic stroke cases recorded during the COVID-19 and pre-COVID-19 pandemic periods.

	Pre-COVID-19 (*n* = 297)	COVID-19 (*n* = 275)	*p* Value
Subtype, *n* (%)			0.037
LAA	126 (42.4)	109 (39.6)	
SVO	118 (39.7)	100 (36.4)	
CE	22 (7.4)	41 (14.9)	
Other determined	4 (1.3)	7 (2.5)	
Undetermined	27 (9.1)	18 (6.5)	
Acute treatments, *n* (%)			0.278
None	261 (87.9)	175 (63.6)	
IV	7 (2.4)	5 (1.8)	
IA	23 (7.7)	17 (6.2)	
IV and IA	6 (2.0)	13 (4.7)	
Time to IV/IA, minutes			
Door-to-IV (*n* = 13:18)	44.0 (35.0–53.0)	43.0 (36.5–53.5)	0.836
Door-to-IA (*n* = 27 ^a^:29)	76.0 (55.0–120.0)	85.0 (64.75–105.0)	0.883

COVID-19—coronavirus disease; LAA—large-artery atherosclerosis; SVO—small-vessel occlusion; CE—cardioembolism; IV—intravenous; IA—intra-arterial. ^a^ Two intra-arterial thrombectomy cases with index stroke progression were dropped.

**Table 3:** Demographic and disease-related characteristics of patients with stroke during Period 1 (the regional COVID-19 outbreak) and the first pre-COVID-19 period.

	1st pre-COVID-19 (*n* = 80)	1st COVID-19 (*n* = 97)	*p* Value
Age, years	65.0 (56.0–76.0)	67.0 (60.0–79.0)	0.288
Male, *n* (%)	42 (52.5)	55 (56.7)	0.684
Previous mRS, *n* (%)			0.435
0	73 (91.2)	92 (94.8)	
1	3 (3.8)	2 (2.1)	
2	2 (2.5)	2 (2.1)	
3	2 (2.5)	0 (0.0)	
4	0 (0.0)	1 (1.0)	
5	0 (0.0)	0 (0.0)	
Clear onset type, *n* (%)	65 (81.2)	81 (83.5)	0.846
Time-to-arrival, hours			
Onset-to-arrival (*n* = 65:81)	10.7 (2.3–27.0)	15.5 (3.2–36.1)	0.308
LNT-to-arrival (*n* = 15:16)	22.1 (8.9–35.9)	15.3 (10.3–33.2)	0.953
Admission route, *n* (%)			0.003
Outpatient	4 (5.0)	21 (21.6)	
Emergency department	76 (95.0)	76 (78.4)	
In-hospital	0 (0.0)	0 (0.0)	
Stroke type, *n* (%)			0.061
Ischemic	58 (72.5)	80 (82.5)	
Hemorrhagic	21 (26.2)	13 (13.4)	
Transient ischemic attack	1 (1.2)	4 (4.1)	
Initial NIHSS	2.0 (1.0–4.0)	2.0 (0.0–5.0)	0.710
Vascular risk factor, *n* (%)			
Previous CVA	11 (13.8)	17 (17.5)	0.633
Coronary heart disease	8 (10.0)	7 (7.2)	0.696
Hypertension	49 (61.2)	57 (58.8)	0.856
Diabetes	25 (31.2)	23 (23.7)	0.341
Dyslipidemia	27 (33.8)	74 (76.3)	<0.001
Atrial fibrillation	5 (6.2)	10 (10.3)	0.488
Current smoker	17 (21.2)	29 (29.9)	0.257
Length of stay, days	11.3 (6.6–23.1)	9.2 (5.8–17.6)	0.460
Discharge NIHSS	1.0 (0.0–3.0)	1.0 (0.0–3.0)	0.580
Expired, *n* (%)	4 (5.0)	4 (4.1)	>0.999

COVID-19—coronavirus disease; mRS—modified Rankin scale; LNT—last normal time; NIHSS—National Institute of Health Stroke Scale; CVA—cerebrovascular attack.

## Data Availability

Data for this study are not publicly available owing to the data-sharing regulations of the Korean Stroke Registry. Only authorized researchers can access the dataset.

## Supplementary Materials

The following supporting information can be downloaded at: https://www.mdpi.com/article/10.3390/healthcare10040604/s1, Table S1: Features of ischemic stroke cases recorded during Period 1.
