# Supplementary material for: Behavioral and Disease-Related Characteristics of Patients with Acute Stroke during the Coronavirus Disease Pandemic
Source: Healthcare (Basel). 2022 Mar 23;10(4):604. doi: 10.3390/healthcare10040604 (PMC9026943; doi:10.3390/healthcare10040604)
Supplement: Supplementary file 1 [file healthcare-10-00604-s001.zip › healthcare-1647978-supplementary.pdf]

Table S1. Features of ischemic stroke cases recorded during Period 1.

	**Pre-COVID-19 (*n* = 88)**	**COVID-19 (*n* = 74)**	***p*-Value**
Subtype, *n* (%)			0.085
LAA	42 (47.7)	34 (45.9)	
SVO	34 (38.6)	20 (27.0)	
CE	6 (6.8)	15 (20.3)	
Other determined	1 (1.1)	0 (0.0)	
Undetermined	5 (5.7)	5 (6.8)	
Acute treatments, *n* (%)			0.369
None	73 (83.0)	64 (86.5)	
IV	1 (1.1)	2 (2.7)	
IA	11 (12.5)	4 (5.4)	
IV and IA	3 (3.4)	4 (5.4)	

Abbreviations: COVID-19, coronavirus disease; LAA, large-artery atherosclerosis; SVO, small-vessel occlusion; CE, cardio-embolism; IV, intravenous; IA, intraarterial.
